# An Observational Study on Patients with Acute Limb Ischemia and SARS-CoV-2 Infection: Early and Late Results in Limb Salvage Rate

**DOI:** 10.3390/jcm10215083

**Published:** 2021-10-29

**Authors:** Sorin Barac, Roxana Ramona Onofrei, Petru Vlad Neagoe, Alexandra Ioana Popescu, Stelian Pantea, Andreea Luciana Rață

**Affiliations:** 1 Department of Vascular Surgery, “Victor Babes” University of Medicine and Pharmacy, 300041 Timisoara, Romania; sorinbarac@gmail.com (S.B.); petruvladneagoe@gmail.com (P.V.N.); rataandreealuciana@gmail.com (A.L.R.); 2 Department of Rehabilitation, Physical Medicine and Rheumatology, “Victor Babes” University of Medicine and Pharmacy, 300041 Timisoara, Romania; onofrei.roxana@umft.ro; 3 “Victor Babes” University of Medicine and Pharmacy, 300041 Timisoara, Romania; alexandra_popescu2007@yahoo.com; 4 Department of Surgery, “Victor Babes” University of Medicine and Pharmacy, 300041 Timisoara, Romania

**Keywords:** SARS-CoV-2 infection, acute limb ischemia, amputation-free survival rate

## Abstract

An observational study on 22 patients presenting with acute limb ischemia and SARS-CoV-2 infection, and without any other embolic risk factors, was performed. All patients were classified according to Rutherford classification for acute limb ischemia. The primary goal of this study was to assess the risk of amputation in these patients after revascularization procedures. The secondary goal was to find the correlation between acute limb ischemia (ALI) severity, patient comorbidities, risk of death, and the association of SARS-CoV-2 infection. The patients were treated by open surgery (18 patients—81.81%) or by the means of endovascular techniques (four patients—18.18%). The amputation-free survival rate was 81.81% in hospital and 86.36% at 1-month follow-up. In this study, the presence of SARS-CoV-2 infection did not influence the amputation-free survival rate: it was only the risk factor for the arterial thrombosis and the trigger for the acute ischemic event. The application of the standard treatment—open surgery or endovascular revascularization—in patients with acute limb ischemia and SARS-CoV-2 infection represents the key to success for lower limb salvage.

## 1. Introduction

Since its outbreak in December 2019 in Wuhan, the coronavirus pandemic has affected more than 166,000,000 people and killed almost 3,600,000 worldwide. In Romania, we have had more than 1,400,000 people infected and more than 40,000 deaths [1].

Blood hypercoagulability is common among COVID-19 patients. In these patients, elevated D-dimers levels are consistently reported (and are associated with the disease worsening). Other coagulation abnormalities that lead to life-threatening complications, such as PT and aPTT prolongation, fibrin degradation products increase, and severe thrombocytopenia, are also present [2,3]. Acute limb ischemia is associated with blood hypercoagulability and can have either embolic or thrombotic causes [4].

Arterial thrombosis in COVID-19 patients can have different forms, from blue-toe syndrome to limb-threatening acute limb ischemia. Only a few patients present within the limb-salvageable interval, and most of them need amputation because of severe gangrene [5].

The aim of the study was to assess the risk of amputation in patients with ALI and SARS-CoV-2 infection. We also tried to underline any correlation between ALI severity, patients’ comorbidities, and the association of SARS-CoV-2 infection.

## 2. Materials and Methods

We performed a retrospective observational study on 22 patients with acute limb ischemia (ALI) and SARS-CoV-2 infection.

The inclusion criteria were: presence of SARS-CoV-2 infection and acute event of vascular disease, i.e., acute limb ischemia.

The clinical status of the patients was defined using the Rutherford classification system [6].

SARS-CoV-2 infection was diagnosed with RT-PCR (Reverse-transcriptase polymerase chain reaction) tests [7] for all patients, and they all underwent chest-computed tomography.

All patients underwent preoperative blood tests (consisting in blood count, creatin-phosphokinase (CK), urea, creatinine, LDH, CRP, D-dimers, etc.). Complete medical history was recorded. Computed tomography angiography (CT-Angio) was performed to assess the extension of the arterial lesions, according to standards of care for this pathology. The entire cohort underwent transthoracic ultrasound to investigate signs of embolic risk factors.

The patients were isolated, and all procedures were performed observing the universal caution regarding the SARS-CoV-2 infection, avoiding cross-contamination, and reducing the risk of viral spread.

All participants in the study read and signed an informed consent. The data were collected under GDPR (General Data Protection Regulation) laws. The study had the agreement from the Hospital Ethics Committee, under the EU GCP Directives, International Conference of Harmonization of Technical Requirements for Registration of Pharmaceuticals for Human Use (ICH), and the Declaration of Helsinki.

The surgical treatment was individualized for every patient considering the arterial lesions and the extent of peripheral lesions. They were treated surgically or by the means of endovascular techniques.

Open surgery consisted of Fogarty embolectomy performed in the operating room under loco-regional or local anesthesia, and none of the patients required intubation because of COVID-19 pneumonia. The approach was either from the femoral or popliteal artery. All patients received antibioprophylaxis with 3rd generation cephalosporins. Before clamping the arteries, a bolus of 80 UI/kgc intravenous heparin was administered.

Endovascular procedures consisted in catheter-directed intra-arterial thrombolysis using Merit Fountain Thrombolysis Catheter and Infusion System^®^ (Merit Medical, South Jordan, UT, USA). The access was either femoral or brachial. After the catheter placement, 10 mL of rt-PA^®^ (Recombinant Tissue Plasminogen Activator—Boehringer Ingelheim International GmbH, Ingelheim am Rhein, Germany) was directly infused with the infusion system. After that, 40 mL of continuous perfusion was administered through the intra-arterial catheter at a rate of 1 mL/h for no more than 24 h, and simultaneously heparin (250 UI/mL/h) was administered through the sheath in continuous perfusion. After 24 h, control angiography was performed, and additional procedures were performed when needed.

The revascularization procedure was chosen in relation to anatomical and/or morphological considerations, especially in patients with multiple atherosclerotic deposits; in those patients, endovascular treatment was preferred in order to avoid complications, such as plaque dissection, or in cases where it was impossible to insert the embolectomy catheter.

Major amputation was defined as above-the-knee or below-the-knee, and minor amputation was considered to refer to either toes or metatarsal amputation [8].

Preoperatively, all patients received antiplatelet (Aspenter^®^ 75 mg daily) and anticoagulant treatment (intravenous unfractioned heparin in continuous perfusion). Postoperatively, all patients received low-molecular-weight heparin, 1 mg/kgc twice daily. We initiated Rivaroxaban 20 mg daily after 5 days of low-molecular-weight heparin [9]. At discharge every patient was given Rivaroxaban^®^ 20 mg daily for 30 days, and after that 2 × 2.5 mg/zi Rivaroxaban^®^ and antiplatelet (Aspenter^®^ 75 mg) daily [10].

### Statistical Analysis

Data were analyzed with MedCalc Statistical Software version 19 (MedCalc Software bvba, Ostend, Belgium). Data are presented as mean and standard deviation, median and interquartile range [IQR], number, and percentage.

Paired t tests were performed between pre- and post-treatment differences on the lower limb viability. Differences were considered statistically significant at *p* < 0.05.

Stepwise logistic analysis was performed in order to identify factors associated with death and amputation.

## 3. Results

Before the pandemic, the number of acute lower limb ischemia (ALI) cases confronting the Department of Vascular Surgery was approximately 180/year. During the Emergency State declared by the Romanian Government in 2020, this number decreased below 126. This period was followed by an increase in patients with stage IIb and III Rutherford acute lower limb ischemia.

Twenty-two patients aged 43–86 years old (mean age 64.91 ± 9.57 years) were admitted into the Vascular Surgery Department of “Pius Brînzeu” Emergency County Hospital in Timișoara were included in this study. None of the patients presented with atrial fibrillation or signs of embolic risk factors (transthoracic ultrasound did not reveal any cardiac thrombus). Also, none of the patients had a peripheral arterial disease history (i.e., intermittent claudication or other signs). There were 15 males (68.18%) and seven females (31.81%). Patients’ demographics, comorbidities, and risk factors are presented in Table 1 below. Fourteen patients (63.64%) had no specific symptoms for COVID-19 infection. The other eight patients had mild COVID-19 symptoms, such as dyspnoea and loss of smell and taste.

The patients were classified according to Rutherford stage in IIA (15 patients—68.18%) and IIB (seven patients—31.81%).

Before admission, 19 patients (86.36%) were under antiplatelet treatment, and three patients (13.64%) received antiretroviral medication (Favipiravir, 1600 mg, bid).

The ischemia time was between 5 and 34 h with a median time of 18.59 h.

Open surgery was performed for 18 patients (81.81%) under local or loco-regional anesthesia—local anesthesia in seven cases (31.81%) and loco-regional anesthesia—11 patients (50%). The access for open surgery was in 12 cases from the femoral artery (52.17%), in four cases (17.39%) from the popliteal artery, and in two cases (8.69%) from both femoral and popliteal artery.

For the endovascular approach (four patients—18.18%), the access was either femoral (three patients—13.63%) or brachial (one patient—4.54%). After 24 h, we performed control angiography after thrombolysis and additional procedures were needed: superficial femoral plain balloon angioplasty (two patients—9.09%) and one at the level of the initial segment of the posterior tibial artery (one patient 4.54%).

**Figure 1:**
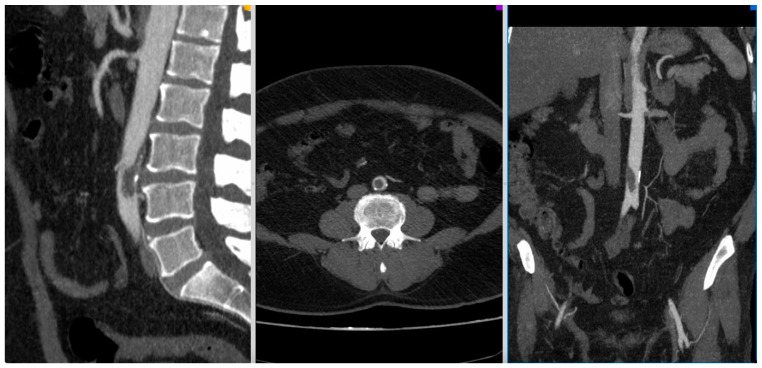
CT-Angio of a 52 yo patient with aortic thrombosis with acute right lower limb ischemia 8 days after SARS-CoV-2 diagnosis.

**Figure 2:**
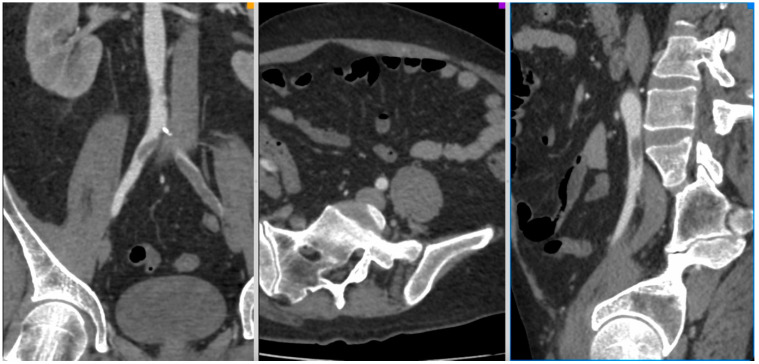
CT-Angio of a 42 yo male patient with aortic and iliac thrombosis with bilateral lower limb acute ischemia IIA Rutherford, 13 days after SARS-CoV-2 diagnosis.

**Figure 3:**
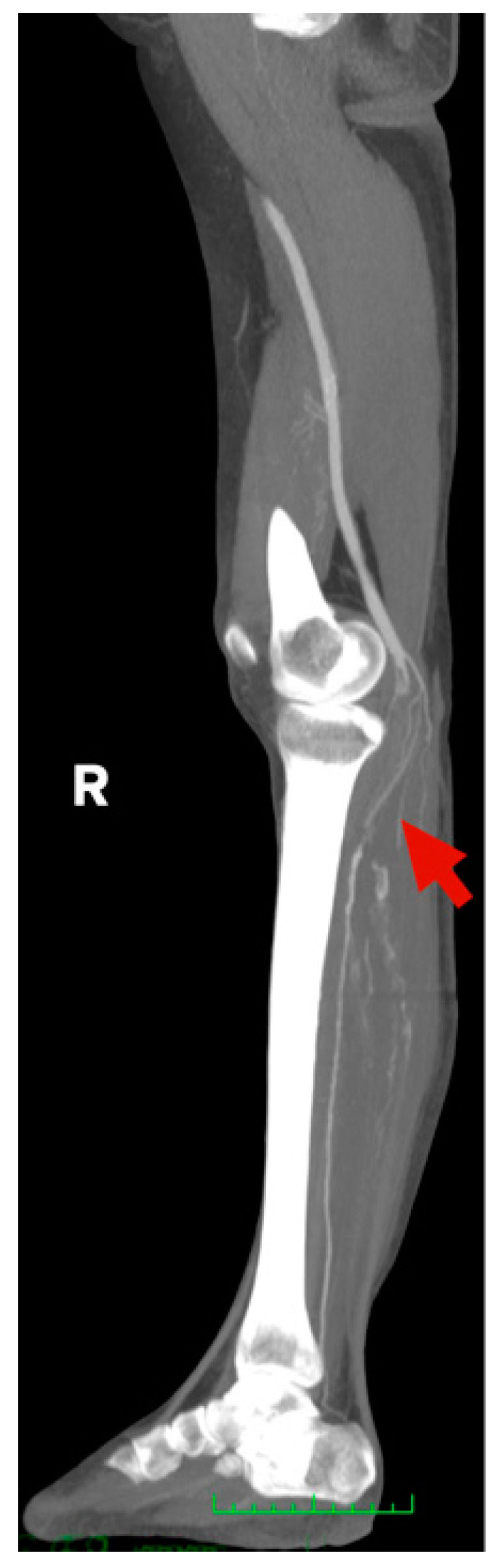
CT-Angio of 58 yo female with stage IIB acute right lower limb ischemia 14 days after SARS-CoV-2 infection.

**Figure 4:**
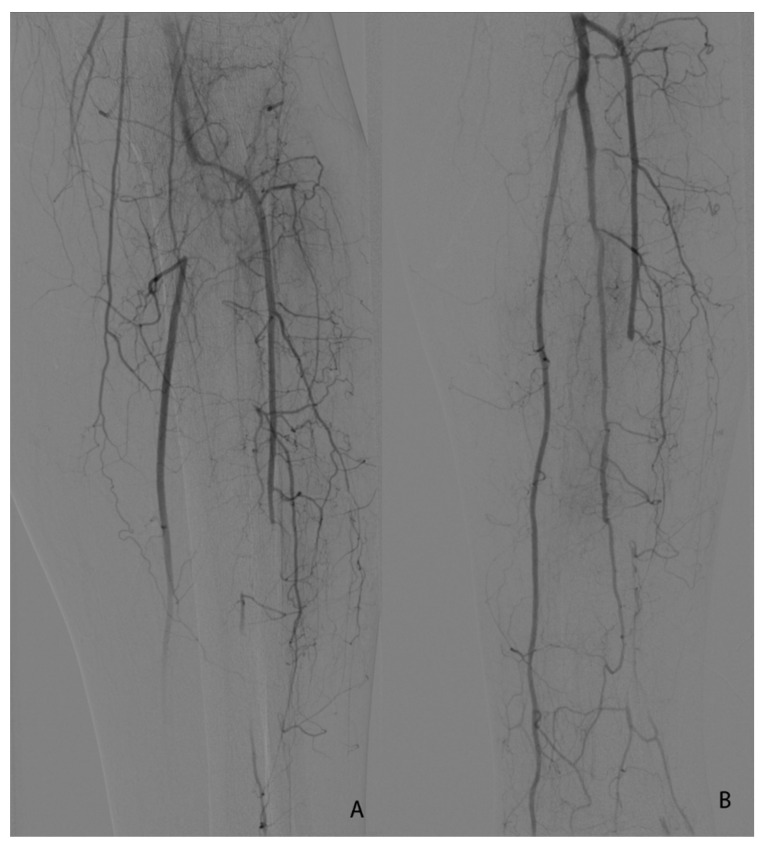
(**A**,**B**). Seldinger angiography of a 64 yo patient with stage IIB Rutherford acute lower left limb ischemia 2 days after SARS-CoV-2 diagnosis. (**A**)—initial image before thrombolysis initiation. (**B**)—final image after balloon angioplasty of the initial segment of the posterior tibial artery.

Figure 1, Figure 2, Figure 3 and Figure 4 show the selected cases from our cohort.

Complications related to surgical procedures were as follows: for the open surgery, one surgical wound infection and one small hematoma (it did not require surgical reintervention); for the endovascular procedures, one small femoral hematoma at the level of the sheath placement (it did not require intervention).

No fasciotomies were performed and no reocclusion was registered in our cohort during hospitalization or for the entire follow-up period.

Three patients (13.63%) died in hospital (because of COVID-19-related causes). The death rate was correlated with high values for ferritin and fibrinogen levels. The causes for mortality are detailed in Table 3.

Two major and one minor amputation were performed during the hospitalization period.

At 1-month follow-up, 18 patients were evaluated (one patient died) and one major amputation was performed. The D-dimers at 1-month follow-up were 490.4 ± 296.5, significantly lower compared to the time of admission (*p* < 0.0001).

The amputation-free survival rate was 72.72% in hospital and 86.36% at 1-month follow-up.

The logistic regression analysis showed that Rutherford IIB was a significant risk factor for amputation in hospital (OR 30, 95% CI 1.29–693.17; *p* = 0.03). No significant risk factors were identified for the amputation at 1-month.

## 4. Discussion

The main finding in our study was the fact that the proper treatment for acute limb ischemia (either open surgery or endovascular) applied in patients with SARS-CoV-2 infection led to higher rates of limb salvage.

The second finding was the fact that prolonged administration of anticoagulant therapy can improve the outcomes of these patients.

All patients suffering from SARS-CoV-2 infection had a high prevalence of thrombotic events [11]. There are a series of articles describing a high prevalence of venous thromboembolism: Klok et al. reported a 31% rate of thrombotic events in a series of 184 critically patients [12] and Helms reported 16.7% rate of pulmonary embolism and 2% vein thrombosis [13].

In patients with no sign of thrombosis at macrovascular level, there were findings at the microvascular level, i.e., small thrombi in pulmonary arterioles [8], superficial dermal vessels, and glomerular capillaries [14,15], and complete luminal thrombosis in small and medium-sized arteries [16].

Acute limb ischemia was reported in patients with moderate COVID-19 symptoms, but there are large studies describing acute limb ischemia as the first symptom of infection. [17,18]

Another aspect is represented by a swift assessment of the demographics of patients with acute limb ischemia. In our study, most of the patients were males aged 60+, findings consistent with those of Cheruiyot, Sanchez and Al-Zoubi [11,19,20].

Other studies showed, at the level of cardiovascular system, modifications such as: acute necrosis, the presence of inflammatory cells, and apoptotic bodies and foci of lymphocytic inflammation [21,22,23,24].

There are three mechanisms involved in vascular thrombosis associated with the presence of SARS-CoV-2: altered vascular wall, abnormal blood flow because of high viscosity, and the hypercoagulable state of the patient.

The relationship between the baseline inflammatory status and the risk of future cardiovascular events is related to CRP values. There are studies stating that CRP is an actual biomodulator of the inflammation within the arterial wall, and that it can alter the behavior of cells in the vessel wall in a way that can promote thrombosis [25]. In our study, the mean values for CRP were 6–8 times higher than normal values.

Fibrinogen, the substrate of thrombin, provides the major meshwork of arterial thrombosis, and high levels have been reported in the presence of SARS-CoV-2 infection [26,27,28]; in these studies, fibrinogen levels were three times normal values and higher values of fibrinogen were found, associated with higher risk of death [29,30,31,32,33]. In our study fibrinogen levels were three times higher than normal values (two of three patients that died had fibrinogen levels three times higher than normal values).

The amputation-free survival rate derives from a proper revascularization treatment by taking into consideration the extent of vascular lesions and the European guidelines in acute limb ischemia in patients presenting within the revascularization window with a viable lower limb [34]. In some studies, the therapeutic indication is correlated both with ischemia time and morphological aspects of the arteries (i.e., atherosclerotic deposits) [35]. In our study, we considered that all of the patients were within the revascularization window (preserved motility and sensitivity of the affected limb) and without any major atherosclerotic deposits (as seen on CTA).

In patients without SARS-CoV-2, amputation rates following acute limb ischemia are between 6% and 23% [36,37]. In this study, the presence of SARS-CoV-2 infection did not influence the amputation-free survival rate: it was only the risk factor for the arterial thrombosis and the trigger for the acute ischemic event.

The recommendations regarding the anticoagulation treatment in patients with ALI and SARS-CoV-2 infection are not well established yet. In a study on 20 patients who underwent revascularization, the usage of systemic heparin was associated with increased survival [38,39]. In this study, all the patients received low molecular weight heparin after revascularization, and none of them required reintervention, which leads to the possibility that heparinization prevents recurrent thrombosis and improves the rate of survival and limb salvage. We chose our anticoagulant therapy based on randomized trials of Rivaroxaban in medically ill patients, which showed the administration of Rivaroxaban after hospital discharge is associated with a significantly lower risk of symptomatic venous thromboembolism [9].

Galyfos et al. [40] conducted a systematic review on 34 articles regarding acute limb ischemia and SARS-CoV-2 infection, involving 540 patients. Mortality rate among these patients was 31.4%, while in our study, the rate was 13.18% at 1-month follow-up; the amputation rate among the 540 patients in the review was 23.2%, compared with 13.18% in this study. In the systematic review by Galyfos et al, the medical treatment was selected in 41.8% of cases, and these had a higher risk of death when compared with any other intervention [40]. With the application of a proper surgical treatment (either open surgery or endovascular) and with a systemic anticoagulant treatment, we think that the patients with acute limb ischemia and SARS-CoV-2 infection who present within the revascularization window can have a good prognosis, despite viral presence as a prothrombotic factor.

### Study Limitations

Our study had several limitations. The first limitation was the small number of patients included in the analysis. Second, because of pandemic limitations, the follow-up period was short. Another limitation was the impossibility of obtaining a real image of the procoagulant status of the patients.

## 5. Conclusions

The application of the standard treatment—open surgery or endovascular revascularization—in patients with acute limb ischemia and SARS-CoV-2 infection is the key to success for lower limb salvage.

The prolonged administration of anticoagulants (both in the periprocedural period and after discharge) can improve surgical results, limb salvage, and patient survival.

## Figures and Tables

**Table 1:** Demographics.

Total No. of Patients	22
Age, years (mean ± SD)	64.91 ± 9.57
Sex	
Male, *n* (%)	15 (68.18)
Female *n* (%)	7 (31.82)
BMI, kg/m^2^ (mean ± SD)	31.63 ± 6.47
**Comorbidities**	
Cardiac insufficiency, *n* (%)	8 (36.36)
Obesity, *n* (%)	16 (72.72)
Diabetes mellitus, *n* (%)	14 (63.64)
Dyslipidemia, *n* (%)	18 (85.71)
High blood pressure, *n* (%)	22 (100)
I, *n* (%)	7 (31.82)
II, *n* (%)	10 (45.45)
III, *n* (%)	5 (22.73)
Chronic obstructive pulmonary disease, *n* (%)	4 (18.18)
Brain cerebrovascular disease, *n* (%)	4 (18.18)
Neoplasm, *n* (%)	2 (9.09)
**Risk factors**	
Smoking, *n* (%)	10 (45.45)
Ischemia time, hours (median [IQR])	18.59 [5–34]
Preoperative antiplatelet treatment, *n* (%)	19 (86.36)
Rutherford classification	
IIA, *n* (%)	15 (68.18)
IIB, *n* (%)	7 (31.81)

The pre-operative blood parameters of the entire sample are presented in Table 2.

**Table 2:** Pre-operative blood parameters.

Characteristic	Range Values	Patients’ Values
Leukocyte count (no. ×10^3^/L), median [IQR] Normal range	4–9.5	8.35 [5.34–14.28]
Neutrophils (%), mean ± SD	45–70%	62.28 ± 12.42
Erythrocyte count (no. ×10^3^/L), median [IQR]	4–5.5	3.64 [3.45–4.24]
Monocyte, median [IQR]	3.5–9%	7.34 [2.89–8.28]
Lymphocyte (no. ×10), median [IQR]	0.8–3.8	1.33 [1.09–1.77]
Hemoglobin level (g/dL), median [IQR]	11.5–15	10.70 [10.31–11.40]
Hematocrit (%), mean ± SD	35–46	34.08 ± 3.47
Platelet count, mean ± SD	150–400	275545 ± 82299
LDH, median [IQR]	120–246	278 [161.3–346.5]
Ferritin level (µg/L), mean ± SD	20–290	728.9 ± 158.5
CRP level (mg/L), mean ± SD	0–10	68.08 ± 23.67
aPTT (s), median [IQR]	25.1–36.5	29.4 [24.4–35.41]
Quick time (s), median [IQR]	9.4–12.5	14.67 [12.68–15.61]
INR, mean ± SD	0.8–1.07	1.27 ± 0.18
VSH (mm/1 h), mean ± SD	1–15	82.41 ± 22.26
AST (U/L), median [IQR]	14–36	23.5 [18–28.25]
ALT (U/L), mean ± SD	0–35	23.91 ± 10.45
D-dimers (ng/mL) mean ± SD	0–243	957 ± 518.6
Urea (mg/dL), median [IQR]	15–36	30 [23–45]
Creatinine (mg/dL), median [IQR]	0.7–1.2	0.89 [0.7–1.42]
Fibrinogen (mg/dL), mean ± SD	200–393	668 ± 168.3
CK (U/L), median [IQR]	30–170	115 [43.75–508.8]

**Table 3:** Mortality causes.

Patient	Gender	Age (yo)	Cause of Death	Complication Related to ALI	Days from Admission	Significant Lab Values at Admission
1	M	59	ARDS	None	18	Ferritin = 980 µg/L
2	F	86	ARDS	None	6	Fibrinogen = 980 mg/dL
3	M	69	ARDS	None	8	Fibrinogen = 879 mg/dL
